# Macrophage Functions in Psoriasis: Lessons from Mouse Models

**DOI:** 10.3390/ijms25105306

**Published:** 2024-05-13

**Authors:** Katarzyna Nazimek, Krzysztof Bryniarski

**Affiliations:** Department of Immunology, Jagiellonian University Medical College, 31-121 Krakow, Poland; katarzyna.nazimek@uj.edu.pl

**Keywords:** autoimmunity, biologics, imiquimod, macrophages, modern dermatological treatment, mouse models, psoriasis, psoriasiform dermatitis, rodent disease models, skin diseases

## Abstract

Psoriasis is a systemic autoimmune/autoinflammatory disease that can be well studied in established mouse models. Skin-resident macrophages are classified into epidermal Langerhans cells and dermal macrophages and are involved in innate immunity, orchestration of adaptive immunity, and maintenance of tissue homeostasis due to their ability to constantly shift their phenotype and adapt to the current microenvironment. Consequently, both macrophage populations play dual roles in psoriasis. In some circumstances, pro-inflammatory activated macrophages and Langerhans cells trigger psoriatic inflammation, while in other cases their anti-inflammatory stimulation results in amelioration of the disease. These features make macrophages interesting candidates for modern therapeutic strategies. Owing to the significant progress in knowledge, our review article summarizes current achievements and indicates future research directions to better understand the function of macrophages in psoriasis.

## 1. Introduction

Since the first description of macrophages by Élie Metchnikoff, many studies have been undertaken to precisely characterize their specific functions. This highly heterogeneous population of innate immune cells plays a key role not only in the first line of defense against pathogens but also in the regulation of inflammation and tissue homeostasis, as well as the induction and orchestration of adaptive immunity. Depending on a wide range of stimuli and the local cytokine milieu, macrophages constantly shift their phenotype to adapt to current tissue conditions [1]. This unique feature of macrophages determines their functional diversity.

Furthermore, monocytes and macrophages together with dendritic cells are often classified as the mononuclear phagocyte system, currently understood to be a dispersed organ physiologically responsible for the clearance of injured, apoptotic, or senescent self-cells, and for the phagocytosis of foreign antigens to induce required immune responses [2]. This additionally emphasizes the duality of macrophage functioning, sometimes as “a killer” and sometimes as “a builder”, depending on the microenvironmental context [3]. Moreover, the pathogenic role of mononuclear phagocytes in psoriasis has recently emerged [4].

In addition to infiltrating the population of monocyte-derived macrophages, they constitute an important group of tissue-resident immune cells that originate from either bone marrow or yolk sac progenitors [5]. Their presence in all body tissues and the high plasticity of their phenotype allow macrophages to participate in both maintaining tissue homeostasis and pathological processes.

### 1.1. Macrophages in the Skin

Skin-resident macrophages can be classified into two main subtypes, i.e., Langerhans cells (LCs) of the epidermis and dermal macrophages [6]. Due to their mixed origin, LCs share the features of both macrophages and dendritic cells [7]. Therefore, they are capable of presenting antigens to T lymphocytes in the skin and draining lymph nodes [6]. At the immunological synapse, LC-derived signaling enables T cells to adapt the effector immune response to the type of antigenic stimulus [8]. Moreover, in certain circumstances, LCs promote local immune tolerance [9]. LC functions and impact on skin homeostasis and immunity have recently been reviewed in detail elsewhere [8,10,11]. However, phenotypic and functional differences allowed the characterization of two distinct subsets of LCs in humans [12], and their similar counterparts can be differentiated in mice [13,14]. In addition, ongoing in-depth genetic analyses are providing new discoveries about LC-like cells [15]. In contrast, dermal macrophages appear to be distinguished by scavenging and phagocytic activities with a low ability to induce T-cell responses [16]. The latter feature seems to make macrophages favoring skin infections even under Th1 lymphocyte control, as shown in cutaneous leishmaniosis [17]. However, while comparing LCs and dermal macrophages, one can assume that LCs are responsible for the activation of CD8+ T lymphocytes while dermal macrophages more often interact with CD4+ T cells [18]. On the other hand, the latter population plays a comprehensive and irreplaceable role in skin homeostasis, including the maintenance of cutaneous salt balance [19].

#### Outlining the Polarization and Classification of Macrophages in Skin Tissue

As mentioned above, the extraordinary plasticity of the current functional phenotype is a hallmark of macrophages. This gives them the opportunity to quickly adapt to the current conditions of the tissue microenvironment and the stimuli acting on them. Attempts to systematize the macrophage polarization process allowed us to distinguish their classical and alternative activation statuses, associated with their pro-inflammatory M1 and anti-inflammatory M2 phenotypes, respectively. However, the already mentioned strict adaptation to the current conditions of the tissue microenvironment causes activated macrophages to show a mixed phenotype. It is no different in the case of skin tissue. However, at steady state, dermal macrophages are more likely to be polarized towards an M2-like phenotype to maintain cutaneous homeostasis. Conversely, stimulated LCs are more susceptible to acquiring an M1-like phenotype, as they provide immune surveillance in the epidermis [20].

Detailed transcriptomic analysis revealed two different subpopulations of dermal CD68+ macrophages that inhabit healthy human skin. While scavenging functions appear to distinguish the Mac1 subpopulation, alternative activation and immunosuppressive properties characterize the Mac2 subtype, closely aligned with fetal macrophages. Remarkably, the latter subpopulation has been shown to predominate in psoriatic skin, which highlights the involvement of fetal macrophage functions, i.e., supporting angiogenesis and leukocyte seeding into tissues, in the pathogenesis of psoriatic inflammation [21]. Furthermore, the tissue microenvironment of aged human skin differs greatly from the physiological conditions, and this has been demonstrated to promote the pro-inflammatory phenotype of dermal macrophages [22]. These findings support the hypothesis that macrophage pro-inflammatory activation may be the main reason for the increasing trend in the incidence of psoriasis with age [23].

Recent comprehensive reviews by Apeku et al. [1] and Xia et al. [24] explored macrophage polarization in the context of skin inflammatory conditions, and highlighted that M1 macrophages predominate in the lesional skin of psoriatic patients and both initiate and drive inflammatory processes, among others, by secreting interleukin-23 (IL-23) and tumor necrosis factor alpha (TNFα). On the other hand, M2 macrophages from non-lesional skin promote the resolution of inflammation and tissue repair.

Nevertheless, both LCs and dermal macrophages are affected in psoriatic patients, and their detailed role in this disease is still a matter of scientific interest.

## 2. Mouse Models of Psoriasis

Psoriasis is an extremely complex systemic inflammatory disease that combines autoimmune and autoinflammatory mechanisms [25]. While helper type 1 (Th1) and Th17 lymphocytes together with γδ T cells are key players in the autoimmune response underlying plaque psoriasis [26], disturbances in the IL-36-dependent pathway are considered crucial triggers of autoinflammation in generalized pustular psoriasis [27]. Therefore, clinical manifestations of psoriasis are very diverse and may vary in individual patients during the course of the disease [28]. The etiopathogenesis of this disease is based on the interaction of autoimmune and autoinflammatory mechanisms, dysbiosis of skin microbiota, and oxidative stress combined with a strong genetic predisposition [29]. The underlying inflammatory processes involve and affect all immune cell populations that inhabit and infiltrate the patient’s skin, and also trigger a systemic inflammatory response, leading to the development of serious complications and comorbidities. Therefore, it is extremely important to study its complex pathogenesis in detail, which in turn will enable the development of modern treatment strategies for particular clinical manifestations. This is possible with the use of different in vivo, ex vivo, and in vitro models, which allow the study of individual immune cells and mechanisms involved in the pathogenesis of psoriasis. So far, the best option for this research is to use an in vivo animal model that best reflects the changes observed in psoriatic patients at the histopathological and molecular levels [30]. Along these lines, Gangwar et al. [31] have elegantly summarized mouse models of psoriasis by category and overlap with human disease, and showed that the total overlap is not greater than 40%. At the same time, the authors predicted the pathways that would be best explored in a specific model. For example, it has been suggested that imiquimod-induced psoriatic inflammation is best suited for studies of the IL-36-dependent pathway.

Among various in vivo mouse models of psoriasis, the most commonly used types include spontaneously developing models as well as those based on genetically modified mice, intradermal administration of cytokines, and epicutaneous administration of imiquimod or xenotransplantation of human psoriatic skin [30,31,32]; their advantages and disadvantages are summarized in Table 1.

### 2.1. Spontaneous versus Genetically-Induced Mouse Models

As summarized elsewhere [30], about a hundred gene mutations that lead to the spontaneous development of psoriasis-like skin lesions in mice have been described so far. These studies, therefore, allow researchers to precisely trace the mechanisms underlying keratinocyte dysfunction and the development of skin lesions. However, the main drawback of these models is the lack of influx of T lymphocytes into the skin, which makes comprehensive studies impossible. On the other hand, the use of transgenic mouse strains allows researchers to focus on the role of a specific gene and its product in the pathogenesis of the disease, making this type of research the most selective, but at the same time the furthest from the complex pathogenesis of the disease.

### 2.2. Human Skin Xenografts

Xenotransplantation requires weakening the mouse immune system, which essentially allows studies to focus on the action of human immune cells that have been transplanted along with the skin. Thus, xenografts reflect the local conditions of the skin affected by psoriatic lesions, while at the same time making it impossible to assess long-term interactions of immune cells at distant tissues together with the systemic inflammatory response. Consequently, this model does not allow for the study of comorbidities. In addition, its technical complexity prompted researchers to develop 3D organoid technology to produce the skin models, which allowed researchers to also study macrophage involvement in psoriasis-related cellular interactions [18].

### 2.3. Inducible Models

Among various cytokines that are intradermally injected into mice to induce psoriasiform dermatitis [31], IL-23 is the most commonly used. Since IL-23-dependent signaling is crucial in the activation of Th17 lymphocytes, one can conclude that this model is best suited for studying the autoimmune background of psoriasis, and macrophages constitute the leading cell population involved in the local production of IL-23 [33]. Conversely, imiquimod acts as an agonist of Toll-like receptors (TLR) type 7 and 8 (TLR7 and TLR8) [34] that induce acute systemic inflammation and thus appear more useful in research on the mechanisms of autoinflammatory reaction. It is worth noting that it was suggested to best reflect the involvement of the IL-36-dependent pathway, as mentioned above [31]. However, in humans, psoriasis is more likely to be associated with a chronic inflammatory response, which may partially limit the direct translation of animal research findings into clinical practice. Furthermore, the course of imiquimod-induced psoriasiform dermatitis varies between mouse strains, and macrophages appear to contribute to these differences [35]. Similarly, macrophage and cytokine involvement varies between imiquimod and mannan-induced models of this disease [36]. Nevertheless, in both inducible models, an effect on macrophage functions is observed, which provides interesting observations of the role of macrophages in psoriasis.

## 3. Macrophage Functions in Mouse Psoriatic Inflammation

The first histological examinations of lesional skin samples from psoriatic patients showed macrophage infiltration in the late 1960s. Thirty years later, researchers provided the first evidence that skin-infiltrating T lymphocytes express a Th1-like phenotype characterized by increased production of interferon gamma (IFNγ) [37]. Since Th1 lymphocyte activation is usually accompanied by macrophage cytotoxicity, delayed-type hypersensitivity was the first mechanism suspected to underlie the pathogenesis of psoriasis [37]. However, a few months later, T lymphocytes from lesional skin of psoriatic patients were shown to express a novel cytokine pattern that distinguished them from Th1 lymphocytes [38]. Subsequent significant progress in research on the pathogenesis of psoriasis allowed the demonstration of the key role of Th17 lymphocytes in the autoimmune reaction while largely neglecting the assessment of macrophage activity. Currently, however, research on macrophages is resuming, and this is where research on animal models of psoriasis comes in handy.

Among the various voices in the debate on the pathogenesis of psoriasis, the involvement of macrophages was confirmed in two independent research models [39], namely, depletion of macrophages in mice with epidermis-specific deletion of inhibitor of nuclear factor kappa B (NF-κB) kinase 2 that develop psoriasis-mimicking skin lesions significantly attenuated skin inflammation. Moreover, in this model, pathogenic macrophage activation was found to be independent of IFNγ [40]. On the other hand, overexpression of the angiopoietin receptor Tie2, mostly in mouse keratinocytes, results in the development of psoriasiform dermatitis with a characteristic infiltration of immune cells, including macrophages, that is closely related to that observed in human lesional skin [41]. Furthermore, activated skin macrophages were identified as an important source of TNFα for psoriatic inflammation in the CD18 hypomorphic (CD18hypo) PL/J mouse model [42]. Later, LCs were identified as the main source of IL-23 that is required to activate IL-17A-producing γδ T cells in an imiquimod-induced mouse model of psoriasis [43]. This model was also applied to prove that activation of resident and monocyte-derived LCs is required to successfully elicit symptomatic psoriatic inflammation [44], and that monocyte-derived LCs are a main source of IL-23 in skin tissue [45]. Production of this cytokine by LCs is stimulated by keratinocytes through signal transducer and activator of transcription 3 (STAT3)-dependent signaling [46]. In addition, IL-17A-exposed keratinocytes impair LC migratory activity [47]. Besides, imiquimod was suggested to activate βKlotho expression in mouse skin macrophages, resulting in TNFα production [48] and thereby contributing to the development of psoriatic inflammation. Moreover, the numbers of LCs and dermal macrophages transiently increase in the late phase of imiquimod-induced psoriatic inflammation [49]. These findings strongly suggest that macrophages are involved in both disease initiation and maintenance. Finally, imiquimod, acting as a TLR7 and TLR8 agonist, can directly activate macrophages and promote their M1 polarization [50] in a 4-1BBL-dependent manner [51], which seems to be pivotal in psoriasis pathogenesis [52].

### 3.1. Polarization of Macrophages in Psoriasis

**Figure 1:**
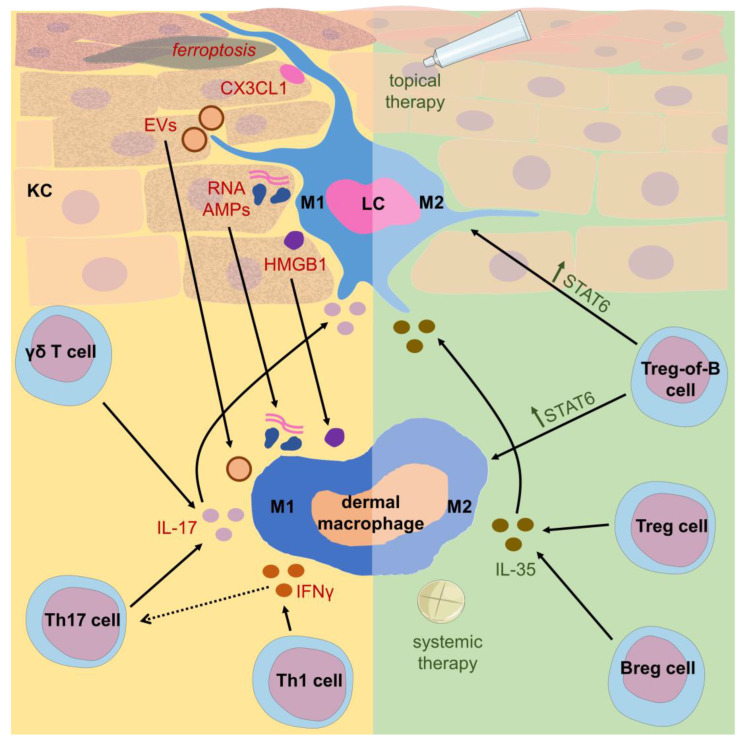
Polarization of Langerhans cells (LC) and dermal macrophages in psoriasis. M1 macrophages play a deleterious role in psoriatic inflammation, while M2 macrophages are able to suppress this reaction. A shift of LCs and macrophages towards a proinflammatory M1 phenotype is induced by keratinocyte (KC) ferroptosis and by KC-released factors like antimicrobial peptides (AMPs), including LL-37-binding self RNA and DNA and triggering macrophage TLR7 and TLR8, high mobility group box 1 (HMGB1) protein recognized as a danger signal, as well as other understudied signals carried by extracellular vesicles (EVs). Furthermore, M1 macrophage infiltration is promoted by CX3CL1 acting via the CX3CR1 receptor. In addition, psoriasis-associated effector cytokines, including IL-17A from Th17 lymphocytes and γδ T cells and Th1 cell-derived IFNγ polarize macrophages towards the M1 phenotype. Conversely, IL-35 secreted by regulatory T (Treg) and B (Breg) lymphocytes together with topically and systemically administered medications promote the macrophage M2 phenotype. Moreover, a specific regulatory cell population, i.e., Treg-of-B cells, was found to increase the expression of STAT6 by macrophages in order to preserve their M2 polarization. Some of the icons were adopted from https://smart.servier.com/ (accessed on 19 April 2024) and used in compliance with the terms of the Creative Commons Attribution 3.0 Unported License.

As mentioned above, psoriatic dermatitis involves inflammatory activation of both LCs and dermal macrophages. Along these lines, M1 polarization appears to predominate among macrophages in human lesional skin [1,24] as well as in imiquimod-induced skin lesions in mice. This model reaction allowed researchers to conclude that the M1 phenotype of macrophages is promoted by high mobility group box-1 (HMGB1), a pro-inflammatory self-protein belonging to danger signals released by inflamed keratinocytes [53]. Moreover, an in vitro assay based on M5-treated HaCaT and THP-1 cell lines also suggested that M1 polarization is stimulated by crosstalk between keratinocytes and macrophages mediated by extracellular vesicles (EVs) [54]. Finally, keratinocyte ferroptosis was shown to promote macrophage M1 polarization [55]. Thus, one can speculate that macrophage polarization towards the M1 phenotype is secondarily stimulated by activated keratinocytes (Figure 1). In addition, IL-17A has been proven to shift macrophages towards the M1 phenotype in mouse skin [56], while C-X3-C motif chemokine ligand 1 and receptor 1 (CX3CL1/CX3CR1) signaling seems to activate the migration of M1 macrophages to the lesional skin of imiquimod-treated mice [57]. Taking into account that macrophage and LC-derived IL-23 activates Th17 lymphocyte differentiation as well as IL-17A production by γδ T cells [43,58], one can conclude that M1-polarized dermal macrophages and LCs are the central cells that drive psoriasis-related autoimmune reaction (Figure 1). Furthermore, stimulation of macrophage glucocorticoid receptors by corticosterone was shown to drive M1 polarization by upregulating STAT1 phosphorylation, while blockage of this signaling alleviated psoriasiform skin inflammation in imiquimod-treated mice [59]. These observations strongly suggest a deleterious role of M1 macrophages in the exacerbation of psoriasis in chronically stressed patients. Cutaneous macrophage polarization was also found to be modulated by pentraxin 3 [60]. Finally, chronic stimulation of macrophage-inducible C-type lectin (Mincle), a pattern recognition receptor, preserves macrophage M1 polarization in imiquimod-induced psoriasis [61]. However, inflammatory macrophages may also downregulate psoriatic inflammation by releasing itaconate [62], an immunomodulatory metabolite that physiologically regulates metabolic rewiring and effector functions of activated macrophages [63].

#### 3.1.1. Impact of Macrophage Polarization in Other Tissues on Psoriatic Inflammation

Data on the involvement of pro-inflammatory-activated macrophages in other tissues are limited but suggestive. Accordingly, M1 polarized gut macrophages appear to aggravate psoriasiform skin inflammation in imiquimod-treated mice by releasing pro-inflammatory cytokines [64]. Moreover, imiquimod-treated mice have severe gut dysbiosis accompanied by increased macrophage activation, which has been linked to an increased risk and severity of inflammatory bowel disease [65]. Simultaneously, intestinal dysbiosis that induces M1 polarization of macrophages in various tissues seems to explain the increased incidence of psoriasis in obese patients [66]. It is worth noting that, among other components of metabolic syndrome, obesity showed the strongest correlation with disease severity in patients with psoriasis [67]. Furthermore, obesity-related adipose tissue inflammation appears to be one of the most important risk factors for the development of psoriasis [68]. These facts drew scientists’ attention to the relationship between adipokine activity and skin inflammation, especially since it appears to favor the development of psoriatic arthritis [69,70,71], and leptin was suggested to promote osteoclastogenesis and bone erosion [72]. Accordingly, along with adipocytes, M1-polarized visceral adipose tissue macrophages are a key source of adipokines that trigger the subclinical inflammation causing the progression of psoriatic lesions. Moreover, macrophages targeted by adipokines, especially leptin, perpetuate a positive feedback loop of metabolic inflammation that drives psoriatic pathology. Conversely, adiponectin appears to attenuate the pro-inflammatory activity of M1 macrophages in psoriasis, but psoriatic patients are characterized by reduced serum levels of this anti-inflammatory adipokine [73]. Therefore, manipulation of adipokine/macrophage-dependent pathways has great therapeutic potential, and studies in animal models should be undertaken to provide a basis for such treatments.

In addition, the polarization of peritoneal macrophages towards the M1 phenotype is observed in various inflammatory conditions [74], and their exposure to IL-23 results in secretion of psoriasis-related cytokines [75,76]. Furthermore, depletion of peritoneal macrophages ameliorates imiquimod-induced psoriatic dermatitis in BALB/c mice [50], while their transfer to the ear of C57BL/6 or CX3CR1^−/−^ mice before imiquimod application modulates the resulting tissue inflammation [57]. Thus, it would be interesting to assess in detail the role of peritoneal macrophages in mouse models of psoriasis.

#### 3.1.2. Macrophage Polarization towards Anti-Inflammatory M2 Phenotype

Unlike their polarized M1 counterparts, M2 macrophages are thought to downregulate psoriatic inflammation. Accordingly, macrophage expression of the mannose receptor CD206, which is usually attributed to their M2 phenotype, was found to play a downregulatory role in a mouse model of mannan-induced psoriasis and psoriatic arthritis associated with the protective activity of reactive oxygen species (ROS) [77]. However, reduced M2 polarization in lesional skin could result from impaired activity of the response gene to complement 32 (RGC-32) cell cycle regulator [78], and could be likely restored under IL-35 activity [79].

In certain circumstances, LCs have been demonstrated to express programmed death ligand 1 (PD-L1) that allows the downregulation of psoriatic dermatitis induced by imiquimod [80]. This likely results from the inhibition of IL-17-producing γδ T cell activity [81]. Moreover, depletion of LCs in the late phase of imiquimod-induced psoriasis resulted in increased infiltration of neutrophils that could potentially exacerbate tissue inflammation [49]. Altogether, these data strongly suggest that LCs play a dual role in psoriatic inflammation, justifying attempts to therapeutically shift LC activity in the anti-inflammatory direction (Figure 1).

#### 3.1.3. Mixed Macrophage Phenotypes in Psoriasis

**Figure 2:**
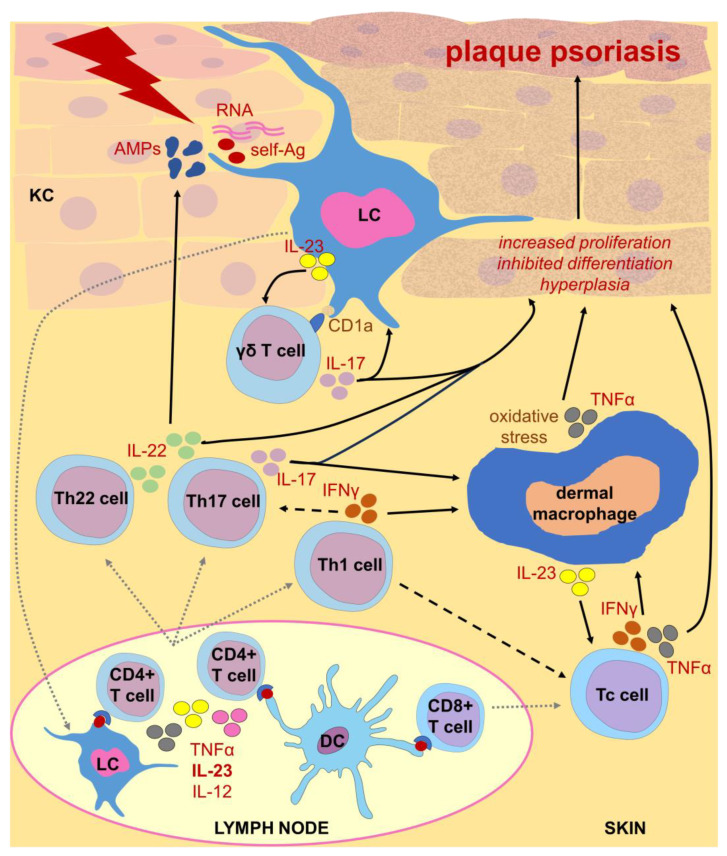
Postulated functions of Langerhans cells (LC) and dermal macrophages in autoimmune responses underlying plaque psoriasis. Psoriasis-inducing triggers stimulate keratinocytes (KCs) to release self-antigens (self-Ag), including self RNA and DNA, as well as antimicrobial peptides (AMPs), particularly LL-37. This drives pro-inflammatory activation of LC that present lipid antigens complexed with CD1a to γδ T cells. The latter cells secrete IL-17A under the influence of LC-derived IL-23. Some activated LCs migrate to draining lymph nodes where they present psoriasis-associated self-Ag to naive CD4+ T lymphocytes, as do dendritic cells (DCs), which are also capable of activating naive CD8+ T lymphocytes. LC- and DC-derived IL-23, IL-12, and TNFα stimulate CD4+ T cell differentiation towards Th17, Th1, and Th22 populations, respectively. Activated T lymphocytes migrate to the dermis where they initiate an autoimmune effector response. Th1 cell-derived IFNγ likely promotes Th17 cell differentiation and cytotoxic T (Tc) cell activity, and mostly stimulates macrophage cytotoxicity accompanied by generation of reactive oxygen species. This is also augmented by Tc cell-derived IFNγ and TNFα, while macrophages drive Tc cell activity through IL-23 release. Simultaneously, IL-22 increases the release of AMPs by KCs. Finally, IL-17A, together with TNFα derived from macrophages and Tc cells and IL-22 derived from Th22 cells, significantly enhances KC proliferation and impairs their differentiation, which leads to epidermal hyperplasia and the development of plaque psoriatic lesions. Some of the icons were adopted from https://smart.servier.com/ (accessed on 19 April 2024) and used in compliance with the terms of the Creative Commons Attribution 3.0 Unported License.

The best example of the previously mentioned mixed macrophage phenotype that is well adapted to the current tissue microenvironment is the M(IL-23) phenotype described by Hou et al. [75]; namely, IL-23-stimulated mouse macrophages were found to produce large amounts of IL-17A, IL-22, and IFNγ due to activation of, respectively, STAT3- and retinoic acid-related orphan receptor gamma T (RORγT)-dependent signaling as well as T-bet-dependent pathway. Surprisingly, the M(IL-23) phenotype can be induced in resting but not in M1/M2 polarized macrophages and differs greatly in the gene expression pattern when compared to M1 and M2 macrophages. Finally, the adoptive transfer of M(IL-23) macrophages significantly exacerbated imiquimod-induced psoriatic inflammation. It is worth noting that IL-23R signaling may induce Th1-like cells [82], and IFNγ may likely induce Th17 cell differentiation [83]. Therefore, these observations suggest that IL-23 induces a unique phenotype in macrophages responsible for their pathogenic activity that drives Th17- and Th1-dependent autoimmune reactions in psoriatic individuals (Figure 2). This hypothesis should be further validated in a mouse model of IL-23-induced psoriasis. Accordingly, preliminary studies demonstrated that the monocyte/macrophage population plays a pivotal role in psoriatic dermatitis induced by intradermal injections of IL-23 into C57BL/6 mice. However, these cells were shown to release large amounts of TNFα rather than IL-17A and IL-22 [84]. One can speculate that these phenotypic discrepancies may be due to differences in macrophage status at the time of IL-23 stimulation, i.e., resting but differentiated versus immature. This again confirms the extraordinary plasticity of the macrophage phenotype.

### 3.2. Macrophage Involvement in Psoriasis-Related Oxidative Stress

From a different point of view, skin macrophages and infiltrating monocytes were suggested to trigger hypersensitivity to itch by releasing pruritic IL-31 [85] and oncostatin M, which enhances neuronal response to histamine and leukotrienes [86]. Furthermore, selective chemical depletion of the small-diameter nociceptors in imiquimod-treated mice to ameliorate skin discomfort and itching was found to reduce the influx of dermal macrophages and subsequent ROS production, which significantly suppressed psoriatic inflammation [87]. The latter observation was very interesting, considering the contribution of oxidative stress to the pathogenesis of psoriasis. The contribution of oxidative stress caused by increased production of ROS and nitrogen species in the development and exacerbation of psoriatic inflammation is now well-established [88,89,90]. Macrophages are one of the main cellular sources of overproduced ROS, and, simultaneously, are strongly affected by oxidative stress. For instance, advanced glycation end products (AGEs), formed under oxidative conditions, ligate macrophage receptors, causing their inflammatory overactivation [91]. On the other hand, however, some studies have revealed a protective role of ROS in imiquimod-induced psoriatic dermatitis. Kim et al. [92] demonstrated that elevated levels of ROS promote beneficial regulatory T cell activity by inducing indoleamine 2,3-dioxygenase expression. Moreover, macrophage-derived ROS were shown to suppress mannan-induced psoriasis and psoriatic arthritis in mice [93,94].

### 3.3. Macrophages Drive Cytokine Signaling in Psoriatic Inflammation and Its Complications

When considering macrophage functions in psoriasis, their presence in all body tissues should be taken into account. Macrophage-derived pro-inflammatory cytokines can activate dermal macrophages in an endocrine manner, proving that chronic inflammatory response increases the risk of psoriasis development. Accordingly, TNFα secreted by macrophages activates subsequent macrophages not only locally but also at a distance, which creates a positive feedback loop of psoriatic inflammation [18]. This partly explains why macrophages are involved in the development of psoriasis-related comorbidities [76]. Accordingly, LL-37 antimicrobial peptide has been recently shown to promote low-density lipoprotein (LDL) uptake by macrophages, followed by foam cell formation that promotes atherosclerosis and cardiovascular complications in mice [95]. Moreover, psoriasis-related increase in S100A8/A9 expression by endothelial cells and macrophages was suggested to play a role in the development of cardiovascular complications [41]. Conversely, macrophage-derived IL-37 was shown to reduce CXCL8, IL-6, and S100 calcium-binding protein A7 (S100A7) production by stimulated keratinocytes of keratin 14 VEGF-A-transgenic (K14-VEGF-Tg) mice, which suppressed local and systemic inflammatory reaction [96], and proved the immune regulatory role of dermal macrophages under certain circumstances.

**Figure 3:**
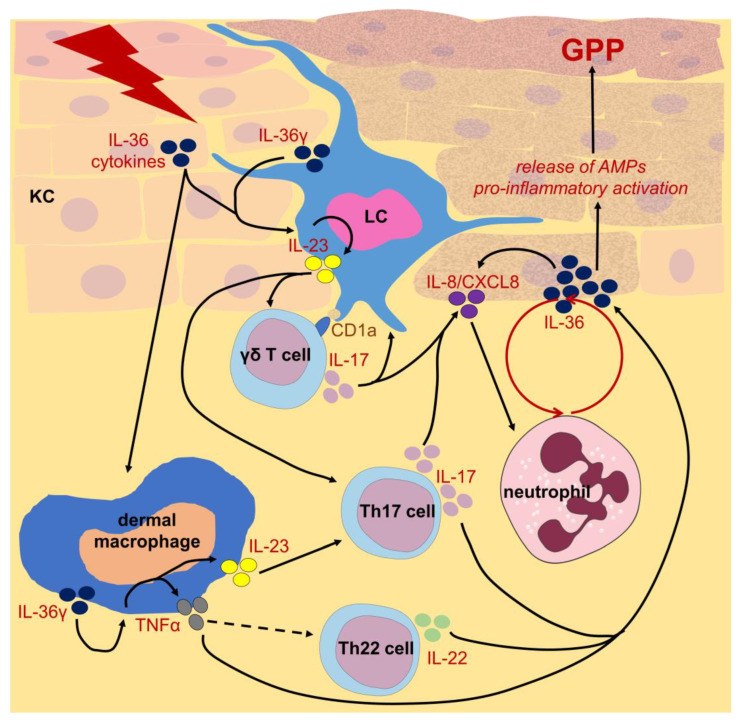
Postulated functions of Langerhans cells (LCs) and dermal macrophages in generalized pustular psoriasis (GPP). GPP-inducing autoinflammatory triggers stimulate keratinocytes (KCs) to release IL-36 cytokine family members, including IL-36γ. After simultaneous secretion by LCs and dermal macrophages, IL-36γ acts in an autocrine and paracrine manner on these cells. As a result, LCs secrete IL-23 that induces IL-17 release by γδ T cells and Th17 lymphocytes. In parallel, dermal macrophages secrete IL-23 and TNFα that activate Th17 and Th22 lymphocytes, respectively. Released IL-17A stimulates the production of IL-8 (CXCL8) by KCs, which drives neutrophil infiltration and activation. Under the influence of neutrophils as well as macrophage-secreted TNFα, Th22 lymphocyte-derived IL-22, and IL-17A, KCs release significant amounts of IL-36 precursors that are activated by KC-derived cathepsin S and neutrophil-derived proteases, such as elastase, cathepsin G, and protease 3, released mostly during the formation of neutrophil extracellular traps. The latter is accompanied by the release of antimicrobial peptides (AMPs) by neutrophils and KCs. A continuously driven IL-36-dependent positive feedback loop leads to the pro-inflammatory activation of KCs and neutrophil accumulation that results in GPP development. Some of the icons were adopted from https://smart.servier.com/ (accessed on 19 April 2024) and used in compliance with the terms of the Creative Commons Attribution 3.0 Unported License.

Additionally, recent reports imply a significant role of LCs and dermal macrophages in the IL-36-dependent signaling cascade underlying the pathogenesis of generalized pustular psoriasis. Macrophages produce IL-36α and IL-36γ, especially. In turn, the latter cytokine activates pro-inflammatory macrophages in an autocrine and paracrine manner. As a result, LCs and macrophages release immune polarizing cytokines, including IL-23 and TNFα, responsible for the induction of Th17 and Th22 lymphocytes as well as γδ T cells that then drive an IL-36-dependent positive feedback loop accompanied by neutrophil accumulation [97] (Figure 3).

Advanced bioinformatic studies followed by validation in imiquimod-treated mice demonstrated that upregulation of ADAM23 metalloprotease in macrophages could be used as a biomarker of psoriasis that is likely involved in its pathogenesis and therefore could also be considered as a predictor of potential targets for biological treatments [98]. Moreover, one can speculate that the expression of ADAM23 enables macrophages to modulate cytokine production by psoriasis-associated effector T cells, as previously shown in the case of dendritic cells [99].

Altogether, both LCs and dermal macrophages play dual roles in psoriasis that depend on current tissue conditions (Figure 1). In some circumstances, pro-inflammatory activated macrophages and LCs exert deleterious effects, triggering psoriatic inflammation (Figure 2 and Figure 3), while in other cases their anti-inflammatory stimulation results in amelioration of disease symptoms. Therefore, modern therapeutic strategies used in psoriasis should take macrophage targeting and modulation into account.

## 4. Macrophages as Therapeutic Targets in Psoriasis

Since inflammatory macrophages play a pathogenic role in psoriasis, restoring their homeostatic functions would be beneficial when considering comprehensive therapy for this systemic disorder.

### 4.1. Therapeutic Balancing of the Macrophage Phenotype

Obviously, skewing macrophage phenotype from M1 towards M2 would produce such beneficial therapeutic effects, and macrophages are one of the direct targets of steroidal drugs used in psoriasis treatment [100]. However, macrophage polarization status can also be modulated by Oxy210, a semi-synthetic oxysterol [101], which opens up the possibility of avoiding long-term steroid treatment and its associated adverse effects. Furthermore, a recent comprehensive review by Xia et al. [24] summarized the signaling pathways that guide macrophage polarization in the context of possible therapeutic targeting. With a focus on Janus-activated kinase (JAK)/STAT, NF-κB, and phosphoinositide 3-kinase (PI3K)/Akt-dependent pathways, it is now important to discover the specific molecular targets whose modulation would provide the most powerful effects.

As summarized above, M2 macrophage polarization is beneficial in preventing and alleviating psoriasis. This can likely be achieved by modulating STAT-dependent pathways under regulatory T (Treg) cell activity. Accordingly, forkhead box p3 (Foxp3)-negative Treg-of-B cells were recently shown to promote M2 macrophage polarization through STAT6 activation in an imiquimod-induced psoriasis model [102]. Moreover, the therapeutic use of non-coding ribonucleic acids (RNAs) and EVs is currently attracting research attention. Accordingly, studies on the circular RNA profile associated with psoriasis identified hsa_circ_0004287 as a candidate for selectively inhibiting macrophage M1 polarization and suppressing imiquimod-induced inflammation in mice [103]. Moreover, engineered EVs produced by fusing the vesicles from annexin A1-overexpressing T lymphocytes with the plasma membranes of M2 macrophages were shown to target macrophages to selectively promote their M2 polarization, which resulted in the alleviation of psoriatic skin inflammation in imiquimod-administered mice [104].

### 4.2. Macrophages as Targets of Immune Regulatory microRNA (miR) Molecules

As reviewed elsewhere [105,106], recent scientific developments have uncovered the pleiotropic role of miR molecules in psoriasis. Most miRs studied to date target an NF-κB-dependent molecular cascade, thereby modulating macrophage activity. Among others, this in turn exacerbates macrophage-driven joint inflammation [107]. Therefore, macrophages seem to be promising targets for RNA-based immunotherapies in psoriasis [108].

Interestingly, our research findings suggest that EV-carried miR-150 released by suppressor T lymphocytes acts on macrophages to diminish their antigen-presenting activity [109,110,111,112,113,114], which in turn suppresses the effector function of Th1 lymphocytes [115,116]. Thus, one can speculate that macrophage targeting with miR-150 would be beneficial in psoriasis treatment, as suggested by our initial results.

### 4.3. Therapeutic Targeting of Macrophage-Dependent Cytokine and Molecular Pathways

**Figure 4:**
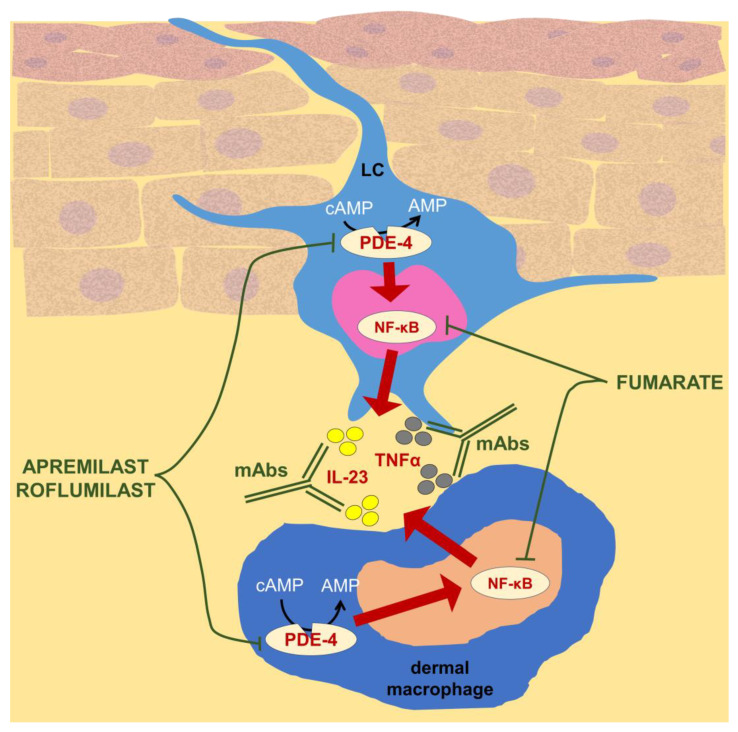
Antipsoriatics that directly affect the activity of Langerhans cells (LCs) and dermal macrophages. Macrophages degrade cyclic adenosine monophosphate (cAMP) into AMP using phosphodiesterase-4 (PDE-4), which activates nuclear factor kappa B (NF-κB)-dependent signaling that leads to the production of pro-inflammatory cytokines, including IL-23 and TNFα. This signaling circuit can be therapeutically inhibited at various stages. Orally administered apremilast and topically applied roflumilast efficiently inhibit PDE-4 activity, while dimethyl fumarate, a first-line systemic therapeutic for plaque psoriasis, blocks the activity of the NF-κB transcription factor, thereby preventing the production of pro-inflammatory cytokines. Finally, various monoclonal antibodies (mAbs) that neutralize specific cytokines have already been approved for the treatment of psoriasis. These include anti-TNFα mAbs such as Infliximab, Adalimumab, and Certolizumab pegol, as well as Ustekinumab, which binds to the p40 subunit shared by IL-12 and IL-23, and mAbs directed against IL-23p19 subunit, e.g., Guselkumab and Tildrakizumab. Some of the icons were adopted from https://smart.servier.com/ (accessed on 7 May 2024) and used in compliance with the terms of the Creative Commons Attribution 3.0 Unported License.

From another point of view, macrophages are an important cellular source of pro-inflammatory cytokines and their active intracellular signaling pathways drive molecular circuits in psoriatic inflammation. Interestingly, some of the already approved anti-psoriatic medications, including biologics and small molecule inhibitors, directly target dermal macrophages and LCs [117,118,119], as summarized in Figure 4. Inhibition of particular cytokine by specific monoclonal antibodies produces promising but transient therapeutic effects, necessitating repeated treatments [120]. Thus, modern therapeutic strategies should simultaneously aim at the inhibition of macrophage secretory activity, which appears to induce durable effects. Along these lines, inhibition of TNFα seems to be the best example of such a dependence. Namely, a better understanding of the pathways that stimulate macrophages to release TNFα allowed researchers to propose several targets, the therapeutic inhibition or activation of which should lead to a permanent reduction in TNFα production [121]. Moreover, etanercept has recently been proposed to promote macrophage M2 polarization [122], which appears to significantly enhance the therapeutic efficacy of this biologic by its dual action, i.e., simultaneous inhibition of TNFα activity and macrophage alternative activation. Additionally, such an approach may also increase the therapeutic efficacy of anti-IL-1β therapy [120], especially since macrophage-released IL-1β fuels psoriatic inflammation by driving IL-17 activity [123]. Finally, therapeutic blockage of IL-23 and IL-17 cytokines has also been suggested to affect monocyte/macrophage activity [124]. The release of pro-inflammatory cytokines by macrophages can also be prevented by targeting the cyclic GMP-AMP synthase (cGAS)-stimulator of interferon genes (STING) pathway [125]. Interestingly, macrophages may likely be therapeutically prompted to degrade pro-inflammatory cytokines. Accordingly, a newly constructed protein degrader for IL-17A was found to be effective in ameliorating imiquimod-induced psoriasis, suggesting that macrophages could be responsible for this assembled IL-17A degradation [126].

It is worth noting that inflammatory macrophages have a reduced wound-healing capacity, which could be deleterious in psoriatic individuals. Thus, restoring this homeostatic macrophage function may be beneficial, and NF-κB appears to be an interesting therapeutic target [127]. In addition, overactivated TLR signaling also contributes to psoriasis development [128], and this could likely be ameliorated by stimulating the serum- and glucocorticoid-regulated protein kinase 1 (SGK1)-dependent pathway in macrophages [129]. It is worth noting that pro-inflammatory macrophages in the skin can be therapeutically targeted by the hyaluronic acid nanoparticles that provide an interesting tool for targeted drug delivery [130]. Moreover, mice depleted of regulatory T cells were found to develop exacerbated psoriatic inflammation associated with an increased influx and activity of macrophages in the skin [131]. Thus, one can conclude that therapeutic reactivation of regulatory T cells would induce a positive effect by reducing macrophage pro-inflammatory activation, likely in an IL-35-dependent manner [79].

Studies on imiquimod-induced psoriasis have revealed that macrophage infiltration in the skin can be reduced by genetic knockdown of the gene encoding the transient receptor potential cation channel subfamily V member 4 (TRPV4) calcium ion channel [132] as well as by orally administered resveratrol [133]. Analogously, at present, skin-infiltrating monocytes have gained researchers’ attention as possible therapeutic targets to prevent their in situ differentiation into inflammatory macrophages or dendritic cells [134]. Such treatment strategies seem to induce longer-lasting effects and therefore could be better tolerated by patients.

As formerly summarized [135], herbal preparations may also exhibit anti-inflammatory effects in psoriasis by acting on macrophages [136] and diminishing their M1 polarization [137], especially when combined with immune suppressive medications [138]. Similar effects may also be induced by *Kaempferia parviflora* extract [139]. Furthermore, traditional medicines may also ameliorate the inflammatory activity of LCs [140]. In addition, LCs are targeted by the topically applied vitamin D analog calcipotriol, which results in the suppression of imiquimod-induced psoriasis [141].

Modifying dietary habits also plays an important supporting role in the treatment of psoriasis. Some studies based on an imiquimod-induced mouse model suggest that yogurt consumption may reduce disease severity by targeting lactate/GPR81 signaling in macrophages [142], while omega-3 polyunsaturated fatty acids inhibit the secretion of pro-inflammatory cytokines by macrophages [143].

Macrophages are likely targeted by dimethyl fumarate approved for systemic therapy of plaque psoriasis (Figure 4). This drug is suggested to efficiently block Th1 and Th17 lymphocyte differentiation [144]. Moreover, as discussed above, macrophages are an important source of inflammatory cytokines in psoriasis and their effector activity is downregulated by cytokine inhibitors [145,146,147,148]. Similarly, macrophages have recently emerged as promising potential targets in attempts to therapeutically block the IL-36-dependent inflammation underlying generalized pustular psoriasis [97].

Furthermore, studies on CD1a-expressing transgenic mice allowed the discovery of the ability of epidermal LCs to present self-lipid antigens and thus trigger psoriatic inflammation. Therefore, CD1a blockage has been proposed as an interesting treatment option for psoriatic individuals [149,150].

## 5. Conclusions and Future Directions

To conclude, research on the involvement of macrophages and LCs in the regulation of psoriatic dermatitis and the accompanying systemic inflammatory response, as well as comorbidities, is extremely valuable for a thorough understanding of the pathogenesis of this disease and the development of new therapeutic strategies. Therefore, future research should be aimed at filling the most clinically relevant gaps in our current knowledge: i. how do LCs and dermal macrophages contribute to psoriasis complications and comorbidities; ii. what is the impact of macrophages activated in other inflamed tissues on the course of psoriatic inflammation; iii. how do anti-psoriatic drugs affect macrophage functions; and iv. how to induce a stable anti-inflammatory phenotype of macrophages at the genetic/molecular level, e.g., by developing a strategy based on RNA interference. Observations from studies involving laboratory animals are extremely helpful in these cases, although one must always bear in mind possible discrepancies in the pathomechanisms of this disease between mice and humans.

## Figures and Tables

**Table 1:** Pros and cons of specific mouse models of psoriasis.

Mouse Model	Strengths and Advantages	Limitations and Disadvantages
spontaneous	best reflects the keratinocyte pathology (e.g., hyperkeratosis), allows the study of cell migration to the skin	difficulties in investigating T cell involvement and drug activity
genetically-modified strains	allows for detailed analysis of the role of individual genes and their products	does not reflect the polygenic basis of the disease and requires the use of expensive mouse strains
induced with cytokines (e.g., IL-23)	enables detailed analysis of the role of a given cytokine and the downstream signaling pathway involving targeted immune cell populations, with little impact on animal welfare	limited to activation of a given signaling cascade and high costs
induced with imiquimod	allows for detailed and multi-aspect analysis of pathomechanism at low costs and ease of use	induction of acute rather than chronic inflammation, with a transient but serious impact on animal welfare
xenotransplantation	accurate reflection of the condition of the patient’s skin tissue, enabling assessment of the effectiveness of drug candidates	no possibility of inducing systemic inflammation, with a serious impact on animal welfare
